# The Correlations of Clinical and Biochemical Indices of Vitamin D with Erectile Dysfunction

**DOI:** 10.25122/jml-2020-0009

**Published:** 2020

**Authors:** Ion Dumbraveanu, Pavel Banov, Iurie Arian, Emil Ceban

**Affiliations:** Department of Urology and Surgical Nephrology, Nicolae Testemiceanu State Medical and Pharmaceutical University, Chisinau, The Republic of Moldova

**Keywords:** Erectile dysfunction, vitamin D, endothelial dysfunction

## Abstract

Erectile dysfunction is a multifactorial disease; it has been demonstrated that endothelial dysfunction plays an essential role in the pathogenesis of this disease, and Vitamin D deficiency is considered to favor endothelial lesions.

Our study, based on a group of 58 patients who have erectile dysfunction and a control group of 26 healthy subjects, tends to confirm that low levels of vitamin D could potentiate the severity of erectile dysfunction, promoting endothelial dysfunction.

Statistical analysis using the Pearson’s correlation criteria showed a robust and significant correlation between vitamin D levels and erectile dysfunction severity (ρ=0.752, p<0.000) according to the SHIM (Sexual Health Inventory For Men) questionnaire. Also, in patients with erectile dysfunction, there is a strong association between vitamin D and testosterone levels (ρ=0.728, p<0.000). At the same time, a negative correlation between vitamin D and BMI (ρ=-0.517, p<0.000); cholesterol (ρ=-0.560, p<0.001), and triglycerides(ρ=-0.529, p<0.005) was observed. Also, a moderate correlation between erectile dysfunction severity degree and testosterone levels (ρ=0.544) was also detected, and the same severity parameter of erectile dysfunction correlates negatively with cholesterol levels (ρ=-0.534). In its turn, the testosterone level correlates negatively with other biochemical indices: cholesterol (ρ=-0.694) and triglycerides (ρ=-0.670).

Vitamin D level reduction, concomitantly with decreased testosterone and increased cholesterol, contributes to the development and maintenance of erectile dysfunction, more probably through endothelial mechanisms. The assessment of vitamin D values can be used as an independent marker in erectile dysfunction assessment. Thus, one of the diagnostic tests recommended for erectile dysfunction should be the determination of the vitamin D serum level.

## Introduction

According to the recommendations of the Fourth International Consultation on Sexual Medicine (ICSM), erectile dysfunction (ED) is defined as man’s constant or recurrent disability to obtain and/or maintain penile erection sufficient to achieve and complete satisfactory sexual intercourse [1].

The first reference statistical data estimated that in 1995, there were more than 152 million men worldwide who presented ED, but a prevalence of approximately 322 million men with ED, an increase of nearly 170 million is presumed for 2025 [2].

According to other data, ED prevalence is about 52% in men aged 40-70, 17% having a mild form, 25% a moderate form, and 10% a severe form [3]. According to a study carried out at the Public Health Management School of “Nicolae Testemitanu” SUMPh, the general prevalence of ED in the Republic of Moldova was 47.1% [21].

Two entities of ED, psychogenic (non-organic) and organic, were described. Although it was previously thought that the psychological factor (absence of sexual education, phobias, and presence of situational factors) is the primary cause, the emphasis is on organic factors now [8]. The main risk factors taken into account are smoking, sedentary lifestyle, alcohol abuse, obesity, dyslipidemia. The causes may also be of vascular (atherosclerosis, venous insufficiency), neurological (stroke, spinal cord injuries, herniated disc) and metabolic origin (diabetes mellitus, hypogonadism, hyperprolactinemia). ED may also be induced by some drugs (antidepressants, tranquilizers, non-selective b-blockers, antiandrogens); pelvic organ surgery or radiotherapy can cause damage to the vessels and nerves involved in erection [8].

Although it is generally classified as a vitamin, the biological structure of vitamin D resembles more with that of hormones. Vitamin D is produced in the epidermis after exposure to ultraviolet B light, from solar or artificial sources, and occurs naturally in a small range of foods. The term “vitamin D” is commonly used to refer to cholecalciferol (vitamin D3), ergocalciferol (vitamin D2), or 25-hydroxyvitamin D (25 (OH) D3).

It is noteworthy that both observational and interventional studies established an association between vitamin D levels and endothelial dysfunction [6].

In addition to the role in bone metabolism, other effects have also been described:

• immunomodulation;
• antiproliferative (inhibits the proliferation of keratinocytes and fibroblasts);
• inhibits the production of renin;
• increases myocardial contractility;
• increases insulin production;
• regulation of apoptosis and angiogenesis (regulates genes that control proliferation) [7].

Considering the complex functions of vitamin D, the correlation between its low serological level and the rate of vascular dysfunction was analyzed. Multiple observational studies associate this parameter with cardiovascular diseases, including coronary artery calcification, stroke, and general mortality [9].

Contradictory opinions on the role of vitamin D in the development of erectile dysfunction have been highlighted while analyzing the literature data, and this study, based on our experience, tends to elucidate the accuracy of the respective concepts.

This study’s purpose was to appreciate vitamin D levels in patients with erectile dysfunction and correlations with the main known risk factors (age, level of testosterone, lipid profile, glycemia, and others.

## Materials and Methods

The study was performed from 2016 until 2019 at Andrology Outpatient Department of IMSP SCR “Timofei Moşneaga” Urology Clinic on a group of 84 male patients between 22 to 67 years who complained of sexual or reproductive problems and who agreed to perform vitamin D determination analysis. To put preliminary diagnosis, all the patients completed BSSC-M (Brief Sexual Symptom Checklist: MenS Version), SHIM (Sexual Health Inventory For Men), NIH-CPSI (American Public Health Institute Questionnaires for Diagnosis of Chronic Prostatitis), IPE (Index of Premature Ejaculation) questionnaires.

The diagnosis and severity of ED was based on SHIM questionnaire, which is the shortened form of IIEF (International Index of Erectile Function) and contains 5 questions. SHIM score characterizes ED severity of the patient in the following way:

• 22-25: no ED
• 17-21: mild ED
• 12-16: mild to moderate ED
• 8-11: moderate ED
• 5-7: severe ED

Anthropometric examinations including determination of height, weight, body mass index (BMI) were performed in all patients. The presence of known risk factors contributing to ED (smoking, alcohol, sedentary lifestyle, etc.) was studied.

To determine the presence of organic factors which contribute to erectile dysfunction occurrence in all patients with less than 21 SHIM score, the lipid profile, glycaemia, uric acid and testosterone level were assessed. In patients without ED, these examinations were performed optionally, as recommended by national or international protocols on preliminary diagnosis.

Vitamin D were assessed by Chemiluminescence immunoassay (CLIA) method, and levels < 10 ng/mL considered as deficiency, 10-30 ng/mL – insufficiency, 30-100 ng/mL – optimal values.

Correlations between vitamin D levels, ED presence and its severity, as well as known risk factors (age, lipid profile and testosterone) were made. For statistical processing we used SPSS social science package, variant 21. Due to the asymmetric distribution in those two analysed groups, the data were processed using non-parametric indicators which require neither the average nor the standard deviation: Mann-Whitney test and Pearson correlation. The values p <0.05 were considered statistically significant.

## Results

The mean age of subjects enrolled in the study was 43 years, ranging from 22 to 67 years. ED diagnosis was established in 58 patients out of 84 subjects after erectile function evaluation questionnaires completion. In 26 subjects, erectile function was appreciated as satisfactory, with a SHIM score higher than 21, being diagnosed with other sexual problems (e.g., premature ejaculation, sexual incompatibility, penial curvature, and others). The vitamin D level determined in all subjects was between 16.90 - 45.20 ng/ml, with an average of 23.40 ng/ml, the optimal level being considered >30 ng/ml. The demographic data of subjects included in the study are shown in Table 1.

**Table 1:** Demographic data of the subjects included in the study.

**Index**	n (%)**
**Age, years (mean±SD; min-max)**	**42.96 ± 11.83; 22-67**
≤40 years	34 (40.5)
>40 years	50 (59.5)
**IEEF Score (mean±SD)**	**16.7 ± 5.83**
Severe	5 (6.0)
Moderate	12 (14.3)
Mild-Moderate	27 (32.1)
Mild	14 (16.6)
No ED	26 (31.0)
**Body Mass Index (mean±SD; min-max)**	**26.84 ± 2.96; 22-34**
Normal	27 (32.1)
Overweight	41 (48.9)
Obesity I Degree	16 (19.0)
**Comorbidities**		
Diabetes Mellitus*	3 (3.6) + 12 (14.3)*
Chronic Prostatitis	18 (21.4)
Premature Ejaculation	15 (17.9)
**Tobacco Smoking**	
No	47 (56.0)
<1 pack per day	24 (28.6)
1 pack per day	10 (11.9)
>1 pack per day	3 (3.6)
**Alcohol consumption**	
No	4 (4.8)
Rarely	20 (23.8)
Cognac/vodka	8 (9.5)
Wine	10 (11.9)
Beer	16 (19.0)

Note: * primarily diagnosed with hyperglycemia;

** unless otherwise noted.

Thus, the subjects were divided into two groups, group 1 with ED and group 2 with other sexual problems, but with a preserved erectile function. Only patients with a score of less than 21 were included in group 1.

Comparing the two groups (Table 2), we noticed that there are no significant differences in age (p=0.92) and laboratory parameters: glycemia (p=0.295), cholesterol (p=0.857), testosterone (p=0.787).

**Table 2:** Comparison indices in subjects with and without ED.

**Index**	**ED** **n=58**	**No ED** **n=26**	p*
**Age, years (mean±SD)**	44.47 ± 11.8	39.62 ± 11.43	0.092
**SHIM score**	13.6 ± 4.18	23.6 ± 0.98	0.0001
**Testosteron** **(nmol/L)**	16.1 ± 6.01	16.9 ± 7.07	0.787
**BMI**	26.90 ± 2.97	26.67 ± 3.03	0.759
**Vitamin D** **(ng/mL)**	20.5 ± 2.97	29.8 ± 7.17	0.0001
**Glycemia** **(mmol/L)**	5.18 ± 2.14	5.20 ± 0.68	0.332
**Cholesterol** **(mmol/L)**	5.24 ± 1.44	5.22 ± 0.733	0.857

Note: *p- according to the Mann-Whitney U test for independent samples.

However, significant differences were detected concerning the SHIM score and vitamin D level (p<0.001).

In the patients with ED, the mean SHIM score was 13.6, but an insufficient vitamin D level was noticed in 81.0% (n=47), while a deficiency was detected in 12.06% (n=7) and only 6.89% (n=4) had optimal levels.

In patients with other sexual problems, but without ED, vitamin D deficiency was observed in 61.53% (n=16), while 38.46% (n=10) had optimal levels. Thus, in both groups, vitamin D deficiency is noticed, but with a significantly increased ratio of vitamin D insufficiency instances in the case of the ED group (p=0.001).

**Figure 1:**
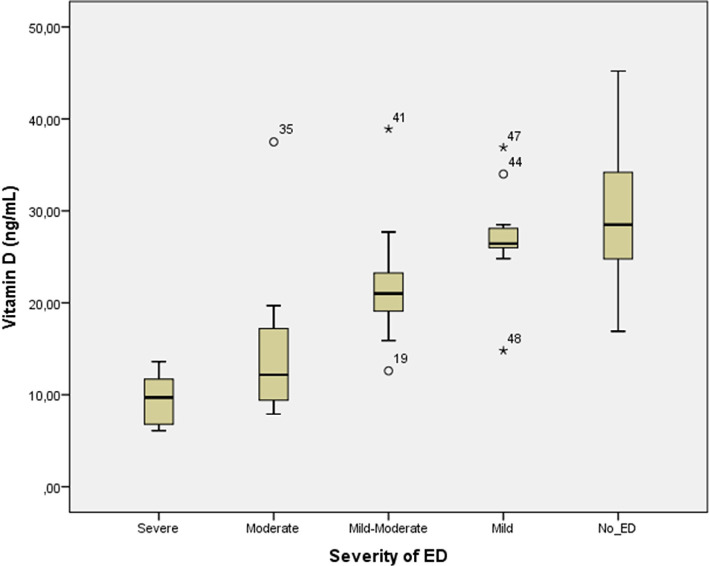
Vitamin D level in subjects with and without ED.

Within the first batch, there is interdependence between ED severity and vitamin D insufficiency degree (Figure 1). Thus, in the group of patients with severe ED (n=5), which is 8.62%, a significantly decreased level of vitamin D is seen, the average being 9.58 ng/ml in comparison with the vitamin D level in the mild ED group, represented by 14 patients (24.13%) - 27.10 ng/ml, in the mild/moderate ED group of 27 patients (46,55%) - 21,70 ng/ml and the moderate ED group of 12 patients (20.68%) – 14,78 ng/ml. Subjects without ED had vitamin D levels close to the optimal minimum level - 29,80 ng/ml.

Vitamin D level assessment according to age (Table 3) showed its progressive decrease with age.

**Table 3:** Vitamin D level according to the age of patients with / without ED.

**Group**		**Age**		
		**<39 years**	**40-49 years**	**>50 years**
**Group 1**	**n**	19	18	21
	**SHIM**	15,84	13,83	10,95
	**Vitamin D, ng/ml**	25,91	18,93	16,99
**Group 2**	**n**	13	8	5
	**SHIM**	>21	>21	>21
	**Vitamin D, ng/ml**	34,26	26,51	23,68

Analyzing the first group of patients, it was observed that in the age group <39 years (n = 19) the mean SHIM score was 15.84, but the mean vitamin D level was 25.91 ng/ml, values that are decreasing in the following groups: 40-49 years with an average SHIM - 13.83, mean VD level - 18.93 ng/ml; >50 years with SHIM average - 10.95, mean VD level - 16.99 ng/ml. Thus, a decrease in VD level and ED severity worsening with age is observed. Also, the link between the same two values regarding ED and VD is noted, both decreasing concurrently. Analogously, the analysis of the second group of subjects reveals a decrease in the VD level with aging. Thus, in the age group <39 years (13 men), the mean VD level is 34.26 ng/ml, values falling in the following groups: 40-49 years with a mean VD – 26.51 ng/ml; >50 years with a mean VD level - 23.68 ng/ml.

**Figure 2:**
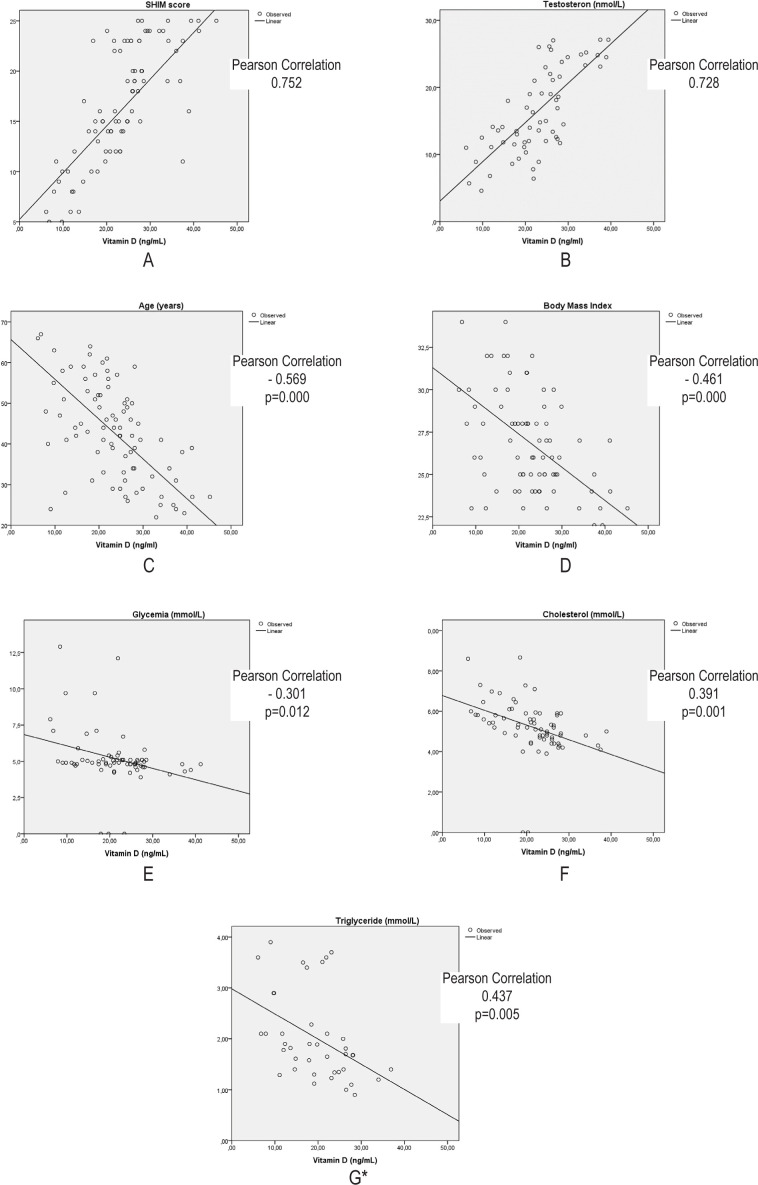
Correlations between the studied indices/parameters and vitamin D (* only for the patients with ED).

Statistical analysis using the Pearson criteria showed a robust and significant correlation between VD level and ED severity (p = 0.752; p <0.001) (Figure 2).

Also, in patients with ED, there is a strong association between VD and testosterone level (p = 0.728, p <0.001). At the same time, a negative correlation between VD and BMI (p = -0.517, p <0.001); cholesterol (p = - 0.560, p <0.001) and triglycerides (p = - 0.529, p <0.005) was observed.

Also, a moderate correlation between ED severity degree and testosterone level (p = 0.544) was detected, and the same severity parameter of ED correlates negatively with cholesterol levels (p = -0.534). In turn, testosterone level correlates negatively with other biochemical indices: cholesterol (p = - 0.694) and triglycerides (p = - 0.670).

Other statistically significant differences regarding other laboratory parameters were not found.

Considering that patients’ age, BMI, and testosterone also strongly correlate with the SCHIM score, these parameters were included as confusion factors in the statistical processing model.

After the statistical analysis, the correlation was recalculated leveling the influence of these factors, and a relatively weaker correlation, but strong and statistically significant (r = 0.601, p<0.001) was obtained, confirming a strong correlation between vitamin D levels and ED.

## Discussion

Although erectile dysfunction is a much-studied topic over the last two decades, opinions on ED genesis remain controversial. Annually, new clinical trials emerge that tend to refine the already existing ED diagnostic and treatment algorithms. For a more coherent interpretation of the obtained results in our study, we wanted to review some of the concepts from the specialty literature and the opinions of the experts in the field.

Several mechanisms can explain endothelial injury in patients with VD deficiency. One of these mechanisms assumes that vitamin D affects the endothelium indirectly by its inhibitory effect on rennin [11]. Consequently, renin lowering reduces the formation of angiotensin II and aldosterone, which leads to a decrease in testosterone levels. If VD is in insufficient amounts, angiotensin II increases, and free radicals increase significantly [12]. These can reduce the production of nitric oxide (NO), which can cause endothelial dysfunctions [13].

A recent study conducted by Kaya C. et al. revealed that endothelial function was impaired in ED patients without any apparent cardiovascular disease or diabetes mellitus. This defective function can be explained by anomalies in the systemic nitric oxide vasodilator system and suggests that ED and vascular disease can be linked to the endothelium level [14].

Another study demonstrated a significant relationship between 25 (OH) D and ED deficiency in male type 2 diabetes patients. This relationship is assumed to be based on an increase in nitric oxide production of 25 (OH) D in endothelial cells through various pathways, inhibiting apoptosis and preventing endothelial dysfunction by preventing the onset of oxidative stress [10].

Direct correlations between ED and various components of the metabolic syndrome, including abdominal obesity, dyslipidemia, hyperglycemia, and high blood pressure, have been reported in several studies [15]. Analyzing this problem, Canguven et al. noted the steady decrease of LDL and TG values in the studied sample, which underwent VD treatment [16].

The influence of this vitamin on the male organism was also analyzed by Weh et al., who demonstrated the direct relationship between androgen levels and VD in 2299 men, so men with high levels of VD also experienced increases in testosterone levels [17].

In our group, the association of low VD with ED was confirmed by patients’ subjective reporting (SHIM Questionnaire), which was confirmed in other recent studies. Farag et al. analyzed 3390 men and reported that deficient VD levels of 520 ng/ml were associated with an increased risk of ED, but the low prevalence of ED was associated with VD levels of 435 ng/ml [18]. When researchers limited their analysis to those 562 men (of 3390 men) with low serum levels of sex hormones, the VD association with ED became even stronger [18].

Another valuable work worth mentioning, elaborated by Proal et al., noticed another mechanism by which VD would influence the male body at a cellular level. Thus, they have shown that in addition to vitamin D nuclear receptor (VDR) activation, 1,25-D displays a high affinity for some of the other body’s nuclear receptors, such as androgen receptors [19].

Therefore, based on the literature data, it is likely that along with other risk factors, even when there are no present clinical symptoms, vitamin D deficiency may be involved in the mechanism that promotes endothelial dysfunction, which ultimately causes ED.

For these reasons, experts in the field suggest routine measurement of vitamin D in ED patients with substitution treatment, if necessary [20].

In fact, most studies concluded that a vitamin D level >30 ng/ml could prevent the negative effect of common ED risk factors such as atherosclerosis and other endothelial dysfunctions.

## Conclusions

In patients with sexual dysfunctions, a VD decrease is more significant in patients with ED. VD level reduction, concomitantly with testosterone levels decrease and cholesterol increase, contributes to the development and maintenance of ED, more probably through endothelial mechanisms. The assessment of vitamin D values can be used as an independent marker in ED assessment. However, additional randomized studies are needed to determine the diagnostic and therapeutic role of vitamin D in patients with erectile dysfunction.

## Conflict of Interest

The authors declare that there is no conflict of interest.

## References

[1] McCabe MP, Sharlip ID, Atalla E (2016). Definitions of Sexual Dysfunctions in Women and Men: A Consensus Statement From the Fourth International Consultation on Sexual Medicine 2015. J Sex Med.

[2] Ayta IA, McKinlay JB, Krane RJ (1999). The likely worldwide increase in erectile dysfunction between 1995 and 2025 and some possible policy consequences. BJU Int..

[3] Feldman HA, Goldstein I, Hatzichristou DG, Krane RJ, McKinlay JB (1994). Impotence and its medical and psychosocial correlates: results of the Massachusetts Male Aging Study. J Urol.

[4] Holick MF, Binkley NC, Bischoff-Ferrari HA, Gordon CM, Hanley DA, Heaney RP, Murad MH, Weaver CM (2011). Evaluation, treatment, and prevention of vitamin D deficiency: an Endocrine Society clinical practice guideline. Endocrine Society. J Clin Endocrinol Metab..

[5] Brewer LC, Michos ED, Reis JP (2011). Vitamin D in atherosclerosis, vascular disease, and endothelial function. Curr Drug Targets..

[6] Tarcin O, Yavuz DG, Ozben B, Telli A, Ogunc AV, Yuksel M, Toprak A, Yazici D, Sancak S, Deyneli O, Akalin S (2009). Effect of vitamin D deficiency and replacement on endothelial function in asymptomatic subjects. J Clin Endocrinol Metab..

[7] Zittermann A, Gummert JF (2010). Nonclassical Vitamin D Action. Nutrients..

[8] Yafi FA, Jenkins L, Albersen M (2016). Erectile dysfunction. Nature reviews Disease primers..

[9] Brondum-Jacobsen P, Nordestgaard BG, Schnohr P, Benn M (2013). 25-hydroxyvitamin D and symptomatic ischemic stroke: An original study and metaanalysis. Ann Neurol.

[10] Basat S, Sivritepe R, Ortaboz D (2018). The relationship between vitamin D level and erectile dysfunction in patients with type 2 diabetes mellitus. Aging Male.

[11] Li YC, Kong J, Wei M, Chen ZF, Liu SQ, Cao LP (2002). 1,25-Dihydroxyvitamin D(3) is a negative endocrine regulator of the renin-angiotensin system. J Clin Invest.

[12] Kawaguchi H, Sawa H, Yasuda H (1990). Endothelin stimulates angiotensin I to angiotensin II conversion in cultured pulmonary artery endothelial cells. J Mol Cell Cardiol.

[13] Balakumar P, Chakkarwar VA, Krishan P, Singh M (2009). Vascular endothelial dysfunction: A tug of war in diabetic nephropathy?. Biomed Pharmacother.

[14] Kaya C, Uslu Z, Karaman I (2006). Is endothelial function impaired in erectile dysfunction patients?. International Journal of Impotence Research..

[15] Esposito K, Giugliano D (2011). Obesity, the metabolic syndrome, and sexual dysfunction in men. Clin Pharmacol Ther.

[16] Canguven O, Talib RA, El Ansari W, Yassin DJ, Al Naimi A (2017). Vitamin D treatment improves levels of sexual hormones, metabolic parameters and erectile function in middle-aged vitamin D deficient men. Aging Male..

[17] Wehr E, Pilz S, Boehm BO (2010). Association of vitamin D status with serum androgen levels in men. Clin Endocrinol (Oxf).

[18] Farag YM, Guallar E, Zhao D (2016). Vitamin D deficiency is independently associated with greater prevalence of erectile dysfunction: the National Health and Nutrition Examination Survey (NHANES) 2001–2004. Atherosclerosis.

[19] Proal AD, Albert PJ, Marshall TG (2009). Dysregulation of the vitamin D nuclear receptor may contribute to the higher prevalence of some autoimmune diseases in women. Ann N Y Acad Sci.

[20] Sorenson MB, Grant WB (2012). Does vitamin D deficiency contributeto erectile dysfunction?. Dermatoendocrinol.

[21] Dumbrăveanu I., Ceban E., Banov P. (2018). Lower urinary tract symptoms and erectile dysfunction in men from the Republic of Moldova. Journal of Medicine and Life..

